# Executive Functions and Mood States in Athletes Performing Exercise Under Hypoxia

**DOI:** 10.3389/fpsyg.2022.906336

**Published:** 2022-05-27

**Authors:** Marco Guicciardi, Riccardo Pazzona, Andrea Manca, Alessandra Monni, Laura Francesca Scalas, Federica Perra, Bruno Leban, Silvana Roberto, Gabriele Mulliri, Giovanna Ghiani, Azzurra Doneddu, Antonio Crisafulli

**Affiliations:** ^1^Department of Education, Psychology, Philosophy, Faculty of Humanities, University of Cagliari, Cagliari, Italy; ^2^Department of Mechanical, Chemical and Materials Engineering, Faculty of Engineering and Architecture, University of Cagliari, Cagliari, Italy; ^3^Sports Physiology Lab, Department of Medical Sciences and Public Health, University of Cagliari, Cagliari, Italy

**Keywords:** executive functions, mood states, exercise, cerebral oxygenation, athletes, inhibition and activation systems

## Abstract

Hypoxia can impair cognitive performance, whereas exercise can enhance it. The effects of hypoxia on cognitive performance during exercise appear to be moderated by exercise duration and intensity and by severity and duration of hypoxia and cognitive task. In normal individuals, exercise under hypoxia can evoke adverse post-exercise mood states, such as tension and fatigue. However, little is known about the effects of hypoxia during exercise in trained athletes. The purpose of this study was to investigate how hypoxia affected executive functions and mood states, assessed, respectively, during and post-exercise and to explore the role of motivation moderators, such as inhibition and activation systems (BIS-BAS). Two different sessions of exercise in normoxia and hypoxia (FiO2 13%), each lasting 18 min, were randomly assigned in a counterbalanced order and administered to seventeen male athletes. During exercise bouts, participants performed a mental task (BST) aimed to produce cognitive interference and suppression. Reaction times and accuracy of responses were recorded. After 5 min, all participants completed two questionnaires assessing mood states (ITAMS) and incidence of symptoms potentially related to hypoxia (AMS-C). The results show that hypoxia impairs cognitive performance in terms of slower reaction times, but a high BAS attenuates this effect. Participants with high BAS show an equivalent cognitive performance under hypoxia and normoxia conditions. No effects were found on mood states. Further research is required to investigate the role of BAS, cognitive abilities, and mood states in prolonged hypoxic conditions.

## Introduction

Executive functions refer to the cognitive abilities involved in the initiation, organization and regulation of behaviors. Executive functions play a vital role in everyday life (McMorris et al., 2011) and are involved in goal-directed behavior, such as exercise (Colcombe and Kramer, 2003; Guiney and Machado, 2013). Scientific evidence suggests that, after a session of exercise requiring acute physical effort, athletes tend to score higher on cognitive tests than when they have not exercised (Etnier and Chang, 2009) and the release of catecholamines was called into question to explain how a single bout of exercise affects cognitive performance (McMorris, 2016).

Although exercises performed at moderate intensity can enhance cognitive functions and prevent neurocognitive disorders (Brisswalter et al., 2002; Lambourne and Tomporowski, 2010), the improvement of the executive functions seems weaker when compared with other kinds of training (Diamond and Ling, 2019) and the interplay between physical and cognitive fatigue must be further investigated (McMorris, 2020; O’Keeffe et al., 2021). Moreover, if exercise takes place at altitude, cognitive performance can be negatively affected (Kramer et al., 1993; Virués-Ortega et al., 2004). Increasing altitude and the consequent severity of hypoxia may attenuate oxygen delivery to the brain tissue and that may result in impairment of brain functions and cognitive abilities (de Aquino Lemos et al., 2012; Diamond, 2013; McMorris et al., 2017), such as executive function, attention, episodic memory, and information processing (Roach et al., 2014; Turner et al., 2015).

Hypoxia is believed to impair cognitive performance (Ando et al., 2020) and it is both the consequence and the cause of many acute and chronic diseases, such as hypertension. As hypoxia levels and exposure time increase, reaction time, and error rate also increase in cognitive tasks (Li et al., 2000; Kourtidou-Papadeli et al., 2008). However, accumulating new evidence suggests that moderate hypoxia may have no adverse effects on cognitive function (Davranche et al., 2016; Morrison et al., 2019; Sun et al., 2019). Some scholars even found in sedentary young women that both exercise and short-term severe hypoxia could have beneficial effects on cognitive function (Lei et al., 2019).

The detrimental or beneficial effects of hypoxia on cognitive performance during exercise are primarily dependent on the interaction of moderators, such as exercise duration and intensity, severity, and duration of exposure to hypoxia and cognitive task type (Ando et al., 2020). In hypoxic conditions, such as high altitude, the acute mountain sickness (AMS) varies according to the duration of the exercise: as the exposure time increases, the discomfort experienced increases (DiPasquale et al., 2015). Several studies have shown that exposure to altitude can lead to increased depressive behavior, anger, fatigue, and irritability (de Aquino Lemos et al., 2012). Moreover, different psychological variables were assessed post-exercise executed in normobaric hypoxia, such as affective responses and mood states (Seo et al., 2015), but the results are still controversial. Keramidas et al. (2016) found that exercise in hypoxia leads to increased post-exercise tension and confusion, while many scholars reported an improvement in mood states (Seo et al., 2015) and positive affective responses (Pasco et al., 2011). Thus, some moderator variables, such as motivational ones, could account for these individual differences (McMorris, 2020). Specifically, a recent review evidenced how it is important to understand the interaction between the motivation and performance in athletes through the study of the EEG prefrontal asymmetry, a biomarker of approach and avoidance motivations (Haehl et al., 2022) and largely related in literature with the BIS and BAS motivational traits (for a review, Vecchio and De Pascalis, 2020). BIS and BAS are two neuropsychological and motivational systems that regulate behavior and emotions: the Behavioral Inhibition System (BIS) regulates avoidance motivation, and the Behavioral Activation System (BAS) regulates approach motivation. The BIS is related to negative emotions (e.g., anxiety, fear, sadness) that might be activated by negative environmental cues of possible punishing, novel, or non-rewarding situations (Gray, 1987, 1990; Carver and White, 1994). The activation of the BIS determines inhibition of behavior that may somehow lead to negative or potential painful outcomes. The BAS is at the basis of approach motivated behaviors toward appetitive goals and is related to positive emotions (e.g., elation, happiness, hope) anticipated by rewarding-positive cues in the environment (Gray, 1990; Carver and White, 1994).

In our previous studies (Mulliri et al., 2020; Magnani et al., 2021) a different duration and intensity of exercise, severity and duration of exposure to hypoxia were considered in samples of young healthy males. Using an interference cognitive task (Guicciardi et al., 2019, 2020), the present study aimed to ascertain in a group of highly trained male athletes how acute hypoxia during exercise affects cognitive functions and post-exercise mood states and if these relationships were moderated by the BIS-BAS systems. *Inter alia*, we hypothesized that: (a) compared to normoxic conditions, exercise performed under hypoxia impairs cognitive performances; (b) under hypoxia conditions, participants’ reaction times increase as the number of trials increases; (c) hypoxia condition is associated with more negative mood states; (d) inhibition and activation systems (BIS-BAS) can moderate the relationship between exercise performed under hypoxia-normoxia conditions and cognitive performances.

## Materials and Methods

### Sample

A group of 17 male athletes was involved (age *m* = 30.24; *sd* = 6.63) based on the following criteria: being involved in regular physical endurance training for at least 3 years and for at least 8 h/week; age between 18 and 45 years; absence of any chronic cardiopulmonary, metabolic, or neurological diseases. 14 athletes have completed the protocol while 3 dropped out prematurely. All athletes were screened to exclude some cognitive deficits and to assess initial signs of discomfort, such as acute mountain sickness, after experiencing hypoxia. This study was performed according to the recommendations of the Code of Ethics for Research in Psychology, Italian Association of Psychology and in accordance with the Declaration of Helsinki. The protocol was approved by the ethics committee of the University of Cagliari (UniCA prot. 0073832).

### Measures

The *Acute Mountain Sickness Cerebral* (AMS-C), developed by Beidleman et al. (2007) is a self-reported 11-items inventory, used to evaluate a weighted AMS cerebral factor score. This condition can be present if an individual’s AMS-C score is > 0.7. In the present study, Cronbach’s alpha is AMS-C = 0.95.

The *Behavioral Inhibitions System and The Behavioral Activation System* (BIS-BAS) were developed by Carver and White (1994) to evaluate the behavioral inhibition and activation systems described by Gray (1987, 1990). It is composed of 20 items with a 5-point Likert scale response format (1 = It does not describe me at all, 5 = It completely describes me). The BIS explores anxious anticipation of negative events (7 items). The BAS investigates the reward sensitivity with three factors: BAS Drive that assesses proactive behaviors (4 items, BASd); BAS Reward Responsiveness explores the tendency to be excited by reward opportunities (5 items, BASrr); and BAS Fun Seeking that investigates the tendency to experiment new sensations (4 items, BASfs). The Italian version showed an acceptable internal reliability (Cronbach’s alphas are BASd = 0.68, BASfs = 0.75, BASrr = 0.74, BIS = 0.72) (Leone et al., 2002). In the present study Cronbach’s alphas are BASd = 0.62, BASfs = 0.60, BASrr = 0.69, BIS = 0.82.

The *Bivalent Shape Task* (BST) developed by Esposito et al. (2013) is a simple non-verbal measure of cognitive interference and suppression in which participants must indicate whether a shape in the center of the screen is a square or a circle. Two response targets are provided for each stimulus, one in the shape of a circle and one in the shape of a square. The participant, equipped with a mouse, is asked to click the response target corresponding to the center of the screen stimulus and target color. The stimulus shape is presented in red, blue, or an unfilled black outline; response targets can be presented in red or blue. Three trial types exist: neutral (black or white stimulus); congruent (the stimulus color matches the response target color); incongruent (the stimulus color mismatches the response target color). The response times were recorded in ms.

The *Italian Mood Scale* (ITAMS) was developed by Quartiroli et al. (2017) to evaluate six mood states relevant in sport and exercise: anger, confusion, depression, fatigue, tension, and vigor. The ITAMS is composed of 24 items expressing mood descriptors with a 5-point Likert scale response format (0 = “not at all”, 4 = “extremely”). Each mood state is assessed through six items. In the present study Cronbach’s alphas are anger = 0.74; confusion = 0.83; depression = 0.63; fatigue = 0.91; tension = 0.67; vigor = 0.87.

The *Positive and Negative Affect Schedule* (PANAS) was developed by Watson et al. (1988) is used to investigate positive (PA) and negative (NA) affect. The PANAS consists of a list of 20 adjectives of which 10 refer to PA (e.g., enthusiastic, interested, and determined) and 10 to NA (e.g., afraid, upset, distressed). All items are rated on a 5-point Likert scale response format (1 = “very little or not at all”, 5 = “very much”). We used the Italian version (Terracciano et al., 2003). In the present study Cronbach’s alphas are PANp = 0.76, PANn = 0.86.

The *Rating of Perceived Effort Scale* (RPE) developed by Borg (1982) is used to quantify an individual’s perception of the physical effort of an activity. The scale consists of intervals from 6 (no exertion at all) to 20 (maximal exertion).

The *Trail Making Test* (TMT) developed by Reitan (1955) is a neuropsychological test that evaluates visual search scanning, speed of processing, mental flexibility, and executive functions. The TMT consists of two parts: A and B. TMT-A requires drawing lines sequentially connecting 25 encircled numbers distributed on a paper sheet. During the TMT-B the participant must draw lines alternating numbers and letters. The score on each part represents the amount of time required to complete the task and any errors made. In our study, we used the Italian version (Giovagnoli et al., 1996).

### Procedure

All participants were assessed on two occasions: baseline visit and experimental phase. During the baseline visit, every participant underwent a medical examination with anamnesis, anthropometric measures (height, weight, and body composition) and psychological assessment. All participants performed an exhaustion cardiopulmonary exercise test on a cycle ergometer (Monark 828E, Vansbro, Sweden), to fix the threshold at which to carry out the exercise in the experimental phase. Specifically, the workload at the gas exchange threshold was calculated (Mulliri et al., 2020; Magnani et al., 2021). To exclude previous cognitive deficits and to assess the perceived exertion or potential emotional discomfort produced by the incremental exercise, TMT, RPE and PANAS were, respectively, administered. Finally, BIS/BAS was administered.

The experimental phase started after 7 days from the baseline visit and included two sessions counterbalanced in reverse order: hypoxia and normoxia. Participants did the BST while pedaling in a sitting position on the cycle ergometer. The responses to the BST were provided through a mouse positioned on a table adjacent to the cycle ergometer (dual task). Finally, they cooled down and recovered while sitting on the cycle ergometer for another 6 min. This procedure was done to minimize possible psychological influences from exposure to hypoxia. Lastly, ITAMS and AMS-C were administered to assess, respectively, mood states post-exercise and to exclude that the inhalation of hypoxic gas could have jeopardized the wellbeing of the athletes.

### Data Analysis

We used SPSS software Version 24.0 (SPSS Inc., Chicago, IL, United States) for all statistical analyses. Preliminary checks were done on TMT scores and PANAS scores, respectively, to exclude previous cognitive deficits or strong adverse mood states post-exercise due to incremental test. Then, we conducted repeated measures analysis on AMS-C scores to exclude that the experimental manipulation has produced initial signs of discomfort. After that, we conducted a repeated measure ANOVA with Greenhouse-Geisser correction to assess if the reaction times differ between the hypoxia and normoxia across four conditions. We explored the trend of time reactions over incremental trials through linear regression analyses, and we performed paired sample *t*-tests of time reactions for low vs. high BAS groups across hypoxia-normoxia conditions. Before running the analyses, we preliminarily tested the assumptions. For ANOVA and paired *t*-tests the data were checked for outliers and normal distribution through the Shapiro-Wilk test. We excluded two outliers (3rd quartile + 3*interquartile range 1st quartile–3*interquartile range), trial 1 for BASfs hypoxia neutral condition and trial 23 for BASrr normoxia congruent condition. Our data were normally distributed except for the BIS hypoxia mixed condition (Shapiro Wilk test *p* < 0.000), which was excluded from the final analyses. For linear regression analyses, we tested the assumptions of normality, linearity, homoscedasticity, and the absence of multicollinearity. All assumptions were met. To calculate the *post hoc* power analysis we employed the G*Power 3.1.9.7 (Faul et al., 2009).

## Results

### Descriptive Statistics of Perceived Effort, Executive Functions, and Accuracy

Preliminarily, we calculated the descriptive statistics to evaluate the individual’s perception of the physical effort on activity (RPE), the executive function measured with the TMT, the PANAS to assess the onset of significant negative affect and the accuracy level reached by all participants in performing the BST task. This was evaluated to exclude that an excessive effort, a scarce cognitive function, a momentary negative affect, or a reduced accuracy might invalidate the other results. The individual’s effort perception indicates a medium level of effort (*m* = 16.4; *sd* = 1.4); participants exhibited normal cognitive abilities (TMT_A *m* = 29.8; *sd* = 5.4; TMT_B *m* = 90.1; *sd* = 27.6; TMT_BmA *m* = 72.6; *sd* = 36.4; cut-off TMT_A = 94; TMT_B = 283; TMT_BmA = 187); the average negative affect (*m* = 14.18, *sd* = 3.84) is consistent with the Italian normative data (*m* = 15.0, *sd* = 5.5) of a non-clinical sample (Terracciano et al., 2003); and average accuracy is over 99% for the majority of BST condition (min = 97.6, max = 100).

We further explored whether a momentary discomfort occurred, we calculated a repeated measure ANOVA of AMS-C measures and we did not find any significant difference on hypoxia versus normoxia condition [*F*(1, 13) = 3.4, *p* > 0.05].

### Comparison of the Hypoxia and Normoxia Conditions

The repeated measures ANOVA, with Greenhouse-Geisser correction, showed that the reaction times for the BST Pure Incongruent condition differ between the hypoxia vs. normoxia condition [*F*_(1, 14)_ = 6.797, *p* < 0.05; *post hoc* power = 0.637]. Specifically, a Bonferroni correction *post hoc* analysis evidenced that participants in the BST Pure Incongruent conditions, under hypoxia condition, employed on average 117.3 ms more than normoxia condition (Hypoxia *m* = 942.5; *sd* = 196.7; Normoxia *m* = 825.2; *sd* = 146.9). This difference is statistically significant (*p* = 0.027). Comparison between hypoxia and normoxia across the other BST conditions did not reach significant differences.

Since the number of subjects who completed the study was small, we opted to consider trials (not subjects) as the number of observations. To obtain the observations we averaged the reaction times of each trial across all participants under hypoxia and normoxia conditions (for example, RT trial 1 for hypoxia condition-first observation-was obtained by averaging the reaction time at trial 1 of 1–14 participants under hypoxia condition). We obtained 10 variables: RT of Hypoxia, Normoxia conditions and RT of Neutral, Congruent, Incongruent, and Mixed conditions under hypoxia and normoxia (average of *N* = 25.5 trials’ reaction times for Neutral, Congruent and Incongruent conditions under hypoxia and normoxia and an average of *N* = 74.5 for Mixed condition under hypoxia and normoxia). This method has the advantage of increasing the number of observations and exploring whether reaction times progressively change from the first to the last trial. Conversely, employing subjects as observations we necessarily had to have a single reaction time measure (averaging all reaction times from the first to the last trials), losing the variability across the trials.

Preliminarily, we compared the hypoxia and normoxia conditions in a paired sample *t*-test. We observed that hypoxia and normoxia differ significantly [*t*_(73)_ = 2.948, *p* = 0.004; *post hoc* power = 0.830], the hypoxia condition is characterized by an enhanced reaction time than normoxia condition (Hypoxia *m* = 829.4; *sd* = 95.4; Normoxia *m* = 795.3; *sd* = 83.8).

Secondarily, we compared the four conditions under hypoxia and normoxia. We observed that in a paired sample *t*-test the BST Pure Incongruent condition was significantly different between hypoxia and normoxia [Pure Incongruent *t*_(22)_ = 3.962, *p* = 0.001; *post hoc* power = 0.965].

Specifically, participants in the BST Pure Incongruent conditions, under hypoxia condition, employed on average 94.5 ms more than in normoxia condition (Hypoxia *m* = 903.3; *sd* = 139.2; Normoxia *m* = 808.8; *sd* = 81.50). This difference is statistically significant (*p* = 0.001). Comparison between hypoxia and normoxia across the other BST conditions did not reach significant differences.

Subsequently, to explore whether reaction times progressively changed, we analyzed the trend of time reactions over incremental trials. We hypothesized that, under hypoxia conditions, participants’ reaction times increase as the number of trials increases. After calculating the linear regression, we found the opposite result in both conditions, such that participants’ reaction times reduce as the number of trials increases (see Table 1).

**TABLE 1:** Linear regression between reaction times and trials number.

		B	*SD*	β	t	*p*
RThypoN	(Constant)	1023.1	59.2		17.3	0.000
	TRIAL	−12.7	4.1	−0.546	−3.1	0.006
RThypoC	(Constant)	859.5	35.7		24.1	0.000
	TRIAL	−6.8	2.3	−0.516	−2.9	0.007
RThypoI	(Constant)	1027.8	50.4		20.4	0.000
	TRIAL	−9.864	3.5	−0.512	−2.8	0.011
RThypoM	(Constant)	913.7	21.2		43.2	0.000
	TRIAL	−2	0.5	−0.436	−4.1	0.000
RTnorN	(Constant)	977	49.9		19.6	0.000
	TRIAL	−11.9	3.1	−0.607	−3.8	0.001
RTnorC	(Constant)	848	31.6		26.8	0.000
	TRIAL	−8.2	1.9	−0.644	−4.3	0.000
RTnorI	(Constant)	890.1	29.1		30.6	0.000
	TRIAL	−6.9	2.1	−0.590	−3.4	0.002
RTnorM	(Constant)	864.7	17.2		50.4	0.000
	TRIAL	−1.4	0.4	−0.385	−3.5	0.001

*RT, reaction times; hypo, hypoxia condition; nor, normoxic condition; I, incongruent; N, neutral; C, congruent; M, mixed (incongruent, congruent, and neutral).*

Therefore, although under both hypoxia and normoxia reaction times progressively reduce with the number of trials, we noticed that the hypoxia condition was characterized by slower time reactions at the beginning in comparison to the normoxia condition. That is, subjects under hypoxia condition are slower in responding to the first trials compared to normoxia condition.

Moreover, no significant differences were found through Repeated measure ANOVA comparing each subscale of the ITAMS between the hypoxia vs. normoxia conditions [ITAMS anger *F*_(1, 14)_ = 2.37, *p* > 0.05; ITAMS confusion *F*_(1, 14)_ = 0.75, *p* > 0.05; ITAMS depression *F*_(1, 14)_ = 0.32, *p* > 0.05; ITAMS fatigue *F*_(1, 14)_ = 1.36, *p* > 0.05; ITAMS tension *F*_(1, 14)_ = 0.00, *p* > 0.05; ITAMS vigor *F*_(1, 14)_ = 1.19, *p* > 0.05]. Therefore, the hypothesis that the hypoxia condition is associated with a more negative emotional status was not supported.

### Moderation of Cognitive Performance Conditions by Motivational Systems Under Hypoxia and Normoxia

We observed that the Behavioral Activation Sensitivity (BAS) significantly influenced the BST performance under hypoxia-normoxia conditions. We compared the time reactions from the first to the last trial of the BST task under hypoxia and normoxia conditions. We were interested in exploring differences across high and low BIS-BAS traits, thus we divided participants in two groups for each BIS/BAS subscale. First, we calculated the descriptive statistics of the four BIS-BAS scales, for the high groups, we selected only participants who scored one standard deviation above the mean, for the low groups, we selected only participants who scored one standard deviation below the mean of each BIS-BAS scale (high and low BAS drive; high and low BAS reward responsiveness etc.) (Blood and Blood, 2007; Table 2). After obtaining these two groups for each BIS-BAS scale we averaged the reaction times of each trial across these participants under hypoxia and normoxia conditions following the method described in paragraph 3.2.

**TABLE 2:** BIS-BAS descriptive statistics and number of participants included in the high or low BIS-BAS groups.

	Min	Max	Mean	*SD*	High group *N*	Low group *N*
BASd	2.25	4.25	3.43	0.47	5	6
BASfs	2.25	4.50	3.47	0.61	4	4
BASrr	3.00	5.00	4.38	0.47	5	6
BIS	2.14	5.00	3.31	0.76	4	6

We then compared the two conditions in a paired sample *t*-test. For low BAS groups we observed the same pattern of results that emerged in the previous analyses, such that hypoxia and normoxia significantly differ. Interestingly, for the high BAS groups the difference between hypoxia and normoxia disappears (Table 3).

**TABLE 3:** Paired sample *t*-test of time reactions BST for low vs. high BAS groups across hypoxia-normoxia conditions.

RT BST	Low						High					
**Neutral**	***HYPO mean (sd)***	***NOR mean (sd)***	***t***	***df***	***P***	***Power***	***HYPO mean (sd)***	***NOR mean (sd)***	***t***	***df***	***P***	***Power***
*Basd*	1014.12 (217.01)	880.31 (183.27)	3.60	20	0.002	0.928	810.84 (177.6)	766.3 (146.52)	1.60	23	0.124	0.334
*Basfs*	938.01 (144.37)	803.77 (97.65)	4.66	19	0.000	0.999	758.38 (105.86)	811.96 (205.88)	-1.22	22	0.235	0.050
*Basrr*	934.88 (191.76)	822.77 (135.19)	4.44	21	0.000	0.984	781.72 (137.46)	798.10 (174.51)	−0.41	22	0.684	0.068
*Bis*	889.43 (217.85)	846.66 (184.22)	1.22	23	0.234	0.249	859.18 (159.92)	841.82 (177.08)	0.39	22	0.698	0.066
**Congruent**	***HYPO mean (sd)***	***NOR mean (sd)***	***t***	***df***	***p***	***Power***	***HYPO mean (sd)***	***NOR mean (sd)***	***t***	***df***	***p***	***Power***
*Basd*	863.09 (94.85)	802.15 (91.71)	2.45	22	0.023	0.649	694.58 (115.51)	681.61 (87.88)	0.59	24	0.563	0.117
*Basfs*	815.04 (115.31)	753.45 (74.29)	2.19	23	0.039	0.562	708.19 (149.99)	750.88 (134.98)	−1.50	24	0.146	0.302
*Basrr*	831.11 (118.81)	741.43 (61.44)	3.27	23	0.003	0.897	681.68 (99.12)	685.10 (88.60)	−0.13	23	0.897	0.051
*Bis*	733.67 (124.49)	735.01 (85.49)	−0.06	24	0.953	0.050	770.66 (118.76)	777.01 (134.26)	−0.18	24	0.860	0.053
**Incongruent**	***HYPO mean (sd)***	***NOR mean (sd)***	***t***	***df***	***p***	***Power***	***HYPO mean (sd)***	***NOR mean (sd)***	***t***	***df***	***p***	***Power***
*Basd*	972.65 (161.04)	889.5 (106.46)	2.26	23	0.034	0.581	868.08 (199.68)	732.79 (97.71)	3.43	23	0.002	0.907
*Basfs*	926.32 (131.27)	838.34 (95.23)	2.72	23	0.012	0.741	854.26 (185.45)	777.75 (116.76)	2.11	23	0.046	0.523
*Basrr*	827.75 (120.31)	742.37 (62.64)	3.02	22	0.006	0.896	767.19 (129.39)	748.44 (204.07)	0.42	23	0.681	0.068
*Bis*	928.16 (249.11)	787.34 (86.72)	3.17	23	0.004	0.858	848.14 (127.29)	832.25 (252.87)	0.30	23	0.764	0.061
**Mixed**	***HYPO mean (sd)***	***NOR mean (sd)***	***t***	***df***	***p***	***Power***	***HYPO mean (sd)***	***NOR mean (sd)***	***t***	***df***	***p***	***Power***
*Basd*	940.23 (139.83)	848.41 (88.78)	4.89	64	0.000	0.998	766.66 (116.14)	758.78 (100.15)	0.48	63	0.633	0.075
*Basfs*	875.56 (136.95)	805.97 (86.64)	3.94	69	0.000	0.973	796.25 (134.75)	818.89 (150.44)	−1.05	73	0.297	0.179
*Basrr*	880.72 (141.57)	801.98 (90.18)	4.21	69	0.000	0.986	741.27 (130.98)	769.62 (114.56)	−1.27	73	0.207	0.241
*Bis*	824.83 (133.11)	806.89 (102.95)	0.96	73	0.343	0.156	821.19 (134.97)	813.29 (126.81)	0.34	70	0.737	0.062

**FIGURE 1:**
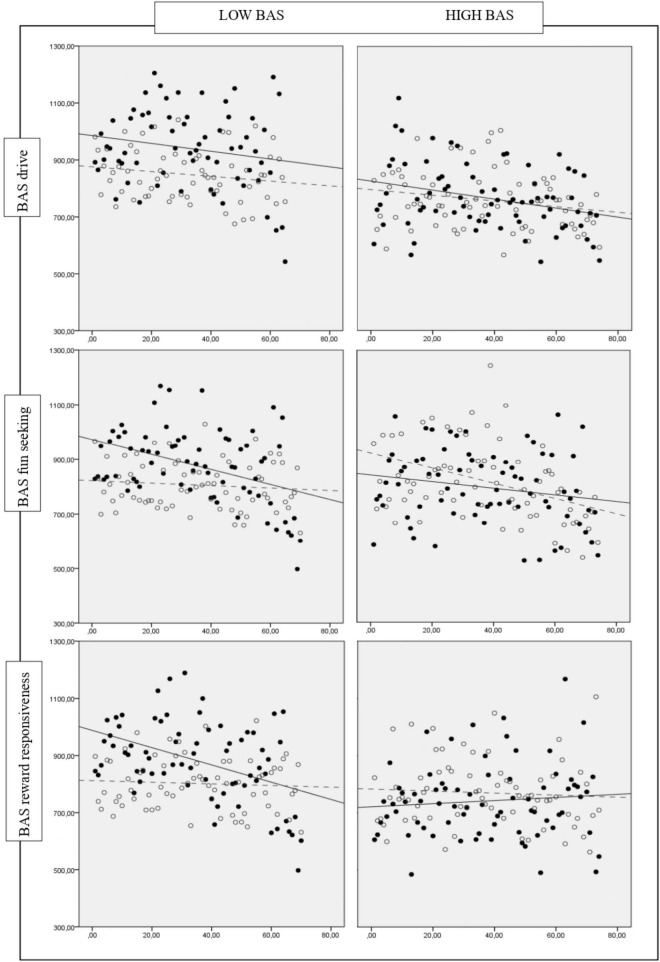
Comparison between the average RT of individuals that perform Hypoxia (black dots and solid line) and Normoxia condition (gray dots and dashed line) across low vs. high BAS groups (drive, fun seeking or reward responsiveness) in the BST Mixed condition. Black dots and solid line, average RT per trials of individuals that perform hypoxia condition; Solid line, trend of average RT on hypoxia condition; Gray dots and dashed line, average RT per trials of individuals that perform normoxia condition; Dashed line, trend of average RT on normoxia condition x, trials numbers; y, time reactions in milliseconds.

Specifically, high BAS participants exhibited an equivalent performance on the Pure Neutral and Congruent conditions under hypoxia and normoxia. In the Mixed condition, we detected a more substantial difference between low BAS groups, which exhibited a different performance in the hypoxia-normoxia conditions, and high BAS groups, which exhibited an equivalent performance (see Figure 1). The Incongruent condition was not affected by high or low BAS.

## Discussion

The most frequent psychological disorders caused by acute physical exercise in a condition of hypoxia are related to the cognitive and emotional aspects. For this reason, monitoring psychological responses in similar situations can represent an indicator of the adverse effects that brain desaturation entails, acting as a predictor of performance.

Currently, the effects of physical exercise in acute hypoxic conditions on cognitive processes and mood states are inconsistent. This could be due to methodological and experimental differences (Jung et al., 2020). The goal of this study was to gain an understanding of the impact of physical exercise in acute hypoxic conditions on cognitive functions and mood states, introducing motivational systems as potential moderators.

The repeated measures ANOVA, with Greenhouse-Geisser correction, showed that participants in the BST Pure Incongruent conditions, under hypoxia condition, exhibited slower RT compared to normoxia condition (on average 117.3 ms more).

Employing trials as observation we confirmed the previous result in a *t*-test corroborating the difference between the hypoxia and normoxia, particularly under Incongruent and Mixed conditions.

Contrary to our second hypothesis, according to which hypoxia progressively worsens reaction times, we found that under both conditions reaction times progressively reduce with trial numbers. However, we noticed that, at the beginning, the hypoxia condition is characterized by slower reaction times in comparison to the normoxia condition. That is, subjects under hypoxia conditions are slower in responding to the first trials compared to normoxia conditions. This paradoxical result can be explained by different factors, such as a learning/habituation effect or by the contribution of cognitive fatigue (McMorris, 2020). As already noted, “individual may predict the sensory consequences during submaximal exercise as being more demanding than in a maximal performance task, where the perceptual demands are less” (McMorris, 2020, p.1706).

Our third hypothesis that the hypoxic condition is associated with more negative mood states was not confirmed. Unlike other studies, we found no effects on mood states. Previous studies have reported in hypoxic conditions an increase in anger, depression and fatigue (Lane et al., 2005), such as an increase in fatigue and a reduction of vigor (Legg et al., 2016) or an increase in tension and confusion when exercise was performed under hypoxic conditions (Keramidas et al., 2016). However, such studies differ for duration and severity of hypoxic conditions and exercise, for environmental conditions where the observations were executed (altitude vs. laboratory) and for the levels of physical skills of participants. For example, Keramidas and colleagues (2016) underwent an exhaustion test in hypoxic conditions, while in our study the athletes inhaled hypoxic gas in a condition parametrized to 70% of the maximum threshold. Seo et al. (2015) hypothesized that the mood state in hypoxia would be impaired following 60 min of hypoxic exposure, but would be improved during the two cycle ergometer exercise intensities (40 and 60% VO2max) and recovery. Based on our previous studies (Mulliri et al., 2020; Magnani et al., 2021) we decided to administer the hypoxic condition for 18 min. Thus, we cannot exclude that increasing the time of hypoxic exposure or reducing the exercise intensities could affect mood states post-exercise such as vigor, fatigue, tension, and confusion.

Our final hypothesis was related to exploring the effect of motivational systems in the relationship between exercise, hypoxia, and cognitive performance. While BIS appeared not involved in this relationship, we observed a relevant effect of BAS. For the low BAS groups, we observed the same pattern of results that emerged in the first analyses, such that hypoxia and normoxia significantly differ. However, for the high BAS groups, the difference between hypoxia and normoxia disappears. High BAS participants exhibited an equivalent performance in the Neutral, Congruent and Mixed conditions under both hypoxia and normoxia. The Mixed condition evidenced the more substantial difference between the low BAS and high BAS groups. The results of individuals with high BAS can be interpreted according to Gray’s conception of individual differences (1990). According to the author, people may have a different sensitivity to BIS and BAS systems. In particular, those with BAS sensitivity tend more frequently to approach potentially rewarding stimuli and their ability to inhibit approach behavior near goals decreases, showing more impulsive behaviors. Therefore, since the BAS is considered an accelerator of approach behavior (Carver et al., 2015), it seems plausible that people with high BAS will show slower reaction times, regardless of the experimental condition, since this is a manifestation of their typical functioning.

The application and generalization of the present study need to be interpreted with caution since only highly trained male athletes were recruited for this investigation. Other limitations are represented by the duration of the exposure to the condition of hypoxia, the low reliability of some subscales, and the small size of the sample that should be increased. In further studies, the role of cognitive fatigue, interoceptive response, physiological correlates, and genetic variations should be deepened to better explain as the inhibition and activation systems moderate the interplay between hypoxia, exercise, cognitive performance, and mood states.

The results of the present investigation could be relevant not only for the application of optimal training protocols or to enhance the performance and safety of recreational and work activities during hypoxia but also from a clinical perspective as it provides insights on the interplay of cognitive, emotional and motivational variables that affect people with chronic diseases during exercise, rendering them hypoxic, such as COPD patients. Future research should investigate the role of BAS, cognitive abilities and mood states in prolonged hypoxic conditions; considering that a longer exposure time leads to an increasing worsening, this methodological change would allow for a more in-depth evaluation.

## Data Availability Statement

The raw data supporting the conclusions of this article will be made available by the authors, without undue reservation.

## Ethics Statement

The studies involving human participants were reviewed and approved by the Ethics Committee UniCa–Prot. n. 0073832 March, 30 2021–(Classif. II/9). The patients/participants provided their written informed consent to participate in this study.

## Author Contributions

AC and MG made the original study design and discussed with the other authors. FP performed the literature analysis. RP and AMa collected the psychological data. SR, GM, GG, AD, and BL collected the biomedical and postural data. AMo performed data analysis. LS conducted the formal analysis. MG, RP, and AMa wrote, reviewed, and edited the manuscript. All authors contributed to the article and approved the submitted version.

## Conflict of Interest

The authors declare that the research was conducted in the absence of any commercial or financial relationships that could be construed as a potential conflict of interest.

## Publisher’s Note

All claims expressed in this article are solely those of the authors and do not necessarily represent those of their affiliated organizations, or those of the publisher, the editors and the reviewers. Any product that may be evaluated in this article, or claim that may be made by its manufacturer, is not guaranteed or endorsed by the publisher.
