# Supplementary material for: Executive Functions and Mood States in Athletes Performing Exercise Under Hypoxia
Source: Front Psychol. 2022 May 27;13:906336. doi: 10.3389/fpsyg.2022.906336 (PMC9196732; doi:10.3389/fpsyg.2022.906336)
Supplement: Supplementary file 1 [file Data_Sheet_1.pdf]

Supplementary Material

1 Supplementary Material

Below we reported some additional information relating to the Italian validations of the two instruments BIS-BAS and ITAMS used to assess the motivational systems and mood states of athletes.

BIS-BAS: The Italian validation of the *Behavioral Inhibitions System and The Behavioral Activation System* (BIS-BAS) was performed by Leone et al. (2002). Authors first analyzed a model of exploratory factor analysis with extraction of four factors and oblique rotation in Mplus, they then tested results through a confirmatory factor analysis. Although the chi-square test was significant, $\chi^2_{(42)} = 94.69$, $p > .001$, approximate fit statistics indicated satisfactory fit: CFI = 0.95, NNFI = .91, RMSEA = 0.05.

ITAMS: The validation of the *Italian Mood Scale* (ITAMS) was performed by Quartiroli et al. (2017). Authors used ESEM analyses, using diagonally weighted least squares with oblique rotation test statistic and WLSMV estimator in Mplus. Although the chi-square test was significant, the approximate fit statistics indicated good fit: $\chi^2_{(147)} = 509.90$, $p < 0.001$, CFI = 0.98, TLI = 0.97, RMSEA = 0.05 [90% CI =0.05,0.06].
